# Dynamic Analysis of Human Natural Killer Cell Response at Single-Cell Resolution in B-Cell Non-Hodgkin Lymphoma

**DOI:** 10.3389/fimmu.2017.01736

**Published:** 2017-12-14

**Authors:** Saheli Sarkar, Pooja Sabhachandani, Dashnamoorthy Ravi, Sayalee Potdar, Sneha Purvey, Afshin Beheshti, Andrew M. Evens, Tania Konry

**Affiliations:** ^1^Department of Pharmaceutical Sciences, Northeastern University, Boston, MA, United States; ^2^Division of Hematology/Oncology, Molecular Oncology Research Institute, Tufts Medical Center, Boston, MA, United States

**Keywords:** dynamic analysis, single cell, lymphoma, non-Hodgkin, microfluidic system, natural killer cell cytotoxicity

## Abstract

Natural killer (NK) cells are phenotypically and functionally diverse lymphocytes that recognize and kill cancer cells. The susceptibility of target cancer cells to NK cell-mediated cytotoxicity depends on the strength and balance of regulatory (activating/inhibitory) ligands expressed on target cell surface. We performed gene expression arrays to determine patterns of NK cell ligands associated with B-cell non-Hodgkin lymphoma (b-NHL). Microarray analyses revealed significant upregulation of a multitude of NK-activating and costimulatory ligands across varied b-NHL cell lines and primary lymphoma cells, including ULBP1, CD72, CD48, and SLAMF6. To correlate genetic signatures with functional anti-lymphoma activity, we developed a dynamic and quantitative cytotoxicity assay in an integrated microfluidic droplet generation and docking array. Individual NK cells and target lymphoma cells were co-encapsulated in picoliter-volume droplets to facilitate monitoring of transient cellular interactions and NK cell effector outcomes at single-cell level. We identified significant variability in NK-lymphoma cell contact duration, frequency, and subsequent cytolysis. Death of lymphoma cells undergoing single contact with NK cells occurred faster than cells that made multiple short contacts. NK cells also killed target cells in droplets *via* contact-independent mechanisms that partially relied on calcium-dependent processes and perforin secretion, but not on cytokines (interferon-γ or tumor necrosis factor-α). We extended this technique to characterize functional heterogeneity in cytolysis of primary cells from b-NHL patients. Tumor cells from two diffuse large B-cell lymphoma patients showed similar contact durations with NK cells; primary Burkitt lymphoma cells made longer contacts and were lysed at later times. We also tested the cytotoxic efficacy of NK-92, a continuously growing NK cell line being investigated as an antitumor therapy, using our droplet-based bioassay. NK-92 cells were found to be more efficient in killing b-NHL cells compared with primary NK cells, requiring shorter contacts for faster killing activity. Taken together, our combined genetic and microfluidic analysis demonstrate b-NHL cell sensitivity to NK cell-based cytotoxicity, which was associated with significant heterogeneity in the dynamic interaction at single-cell level.

## Introduction

Natural killer (NK) cells are innate immune cells that detect and spontaneously kill malignantly transformed cells without prior antigen sensitization (1). The activation of NK cells is regulated by the expression of an array of activating and inhibitory receptors on NK cell surface as well as NK-responsive ligands in target cells (2–6). Determining the expression of regulatory NK cell ligands in target cells, such as cancer cells, is necessary to understand their susceptibility to NK cell-mediated cytotoxicity. However, characterizing target cancer cell molecular profiles may not be sufficient to predict NK cell effector outcomes. Dynamic cell–cell interactions and the effect of environmental factors further increase the level of complexity of these interactions and contribute to the diversity in NK cell function (7–11). This makes individual NK-cancer cell interactions highly heterogeneous (12–14). Potentially, the variability in NK cell effector response at single-cell level could affect overall NK cell-dependent anticancer activity. Thus, analyzing the response of single NK cells, in combination with genetic signatures of target cells, may inform us of the functional diversity and mechanistic differences in NK cell activity. Furthermore, understanding the sensitivity of specific cancer targets to NK cell-mediated cytolysis is a step toward tailoring strategies for NK-based therapeutic applications and optimizing immunomodulatory treatment protocols.

At present, NK cell effectiveness against target cells is evaluated by conventional techniques such as ^51^Chromium release assay, LDH release, and flow cytometry-based analysis (15–21). However, these methods are based on averaging the effects of cell populations and thus not capable of tracking and correlating the outcome of dynamic individual effector–target (E–T) cell interactions. These approaches also do not permit detection of key interactive features, such as repeated scanning, spatial coordination, cooperativity, or mechanism (e.g., contact-dependent vs. independent interactions) of NK cell cytolysis. Here, we report a droplet microfluidics-based technique to quantify variability in NK cell antitumor activity.

Our approach is based on dynamic time-lapse microscopy at single-cell resolution, which has been utilized previously for monitoring individual NK cell interactions with adherent target cells (13, 22–24). However, it is challenging to study heterotypic interaction of suspension cell pairs using standard microscopic methods since one or both cell types can easily drift apart over time. Furthermore, coculture assays of mixed effector and target cells do not allow any control over interaction of individual cells. One effector cell may contact multiple target cells with different dynamics, and *vice versa*. This could potentially affect the response of both effector and target cells and alter the outcome of the assay. In contrast, microfluidic single-cell and cell-pairing bioassays provide improved control over heterotypic cell–cell interaction (25–31). NK cell responses have been investigated at single-cell level in microwells (14, 32–34). However, loading small groups of NK cells (30–50) in microwells could potentially lead to bidirectional homotypic and heterotypic regulation by proximal NK and cancer cells within the same well. Open-configuration hydrodynamic traps and sealed nanowell arrays have been used to measure lytic responses of NK cells at 1:1 E:T (35, 36). These studies showed heterogeneity in kinetic parameters of interaction, migration, and secretion. In the microfluidic droplet array reported here, cells remain effectively trapped at 1:1 E:T in picoliter-volume droplets with minimized signaling and regulatory influences from neighboring NK or target cancer cells. We used this single-cell cytotoxicity imaging assay to assess the dynamics of NK cell responses against B cell non-Hodgkin lymphoma (b-NHL) cells.

There are approximately 80,000 new cases of NHL diagnosed every year in the United States (US), of which >85% are B-cell lymphomas. The b-NHLs are diverse and include a number of subtypes, the most common being diffuse large B cell lymphoma (DLBCL). DLBCL accounts for nearly 30% of newly diagnosed cases of NHL in US. Burkitt Lymphoma (BL) is one of the most proliferative, but rarer, subtypes of b-NHL. Both DLBCL and BL are potentially curable malignancies with combination chemotherapy. However, a portion of patients still endure morbidity and/or die due to the disease or toxicities due to therapy. Given that b-NHL histologic subtypes exhibit prominent genetic heterogeneity (37), we were interested in assessing the molecular profiles of DLBCL and BL, particularly the expression of NK ligands, in primary b-NHL cells and cell lines. Understanding the sensitivity of b-NHL cells to NK cell-dependent cytotoxicity would be beneficial not only for assessing the efficacy of NK cell-based immunotherapeutics against lymphoma cells but also other immunotherapies that rely upon or enhance antibody-dependent cellular cytotoxicity (ADCC) (38–41).

Our genomic analyses showed altered expression of NK-activating and costimulatory ligands in various lymphoma cell lines and b-NHL cells. Our microfluidic droplet-based single-cell assay identified significant heterogeneity in NK-target cell interaction, nature of interactions (contact-dependent vs. contact-independent), involvement of secreted factors, and NK-related lymphoma cell death. We further applied this assay to perform rapid quantification of the variability in responses of patient-derived lymphoma cells. Collectively, our results indicate that this approach is suitable for analyzing the efficacy of therapeutic NK cell lines and cancer targets.

## Materials and Methods

### Cell Isolation and Culture

Raji and SUDHL2, SUDHL-4, and SUDHL10 (B cell lymphoma cell lines) were purchased from American Type Culture Collection (Manassas, VA, USA) and maintained in RPMI-1640 medium supplemented with 10% Fetal Bovine Serum and 1% antibiotic-antimycotic solution (Corning Cellgro, Manassas, VA, USA). NK-92 cells were obtained from Nantkwest, Inc. (Woburn, MA, USA) and maintained in X-Vivo 10 media (Lonza, NJ, USA) supplemented with 5% human serum. All cells were grown at 37°C and 5% CO_2_ in a humidified atmosphere. Cells were routinely passaged every 3 days and harvested at a density of 1 × 10^6^ viable cells/mL.

Primary human CD56^+^ NK cells (≥90% purity) were purchased from Stemcell Technologies (#70037, Cambridge, MA, USA) and cultured overnight in RPMI-1640 media containing 10% FBS, 1% antibiotic and 50 ng/mL IL-2 (Peprotech, Rocky Hill, NJ, USA). Unless otherwise specified, NK cells for different experiments were obtained from different donors. Primary human lymphoma cells were isolated from discarded tissue specimens obtained through Tufts Tumor Repository under IRB exempt protocol. Discarded tissue specimens were minced using disposable 15 mL closed tissue grinder system (Fisher Scientific) and filtered using 30 µM sample pre-separation filter (Miltenyi Biotech, Auburn, CA, USA). Cell suspensions were centrifuged, washed, and re-suspended in RPMI-1640 medium supplemented with 10% fetal bovine serum and 1% antibiotic-antimycotic solution. Enrichment of lymphoma cells was performed by extended cell culture and/or B cell isolation using Easy B cell isolation kit (Stemcell Technologies). The purity of enriched cells were determined by anti-CD20-FITC staining and flow cytometry.

### Transcriptome Analysis

For genome-wide expression profiling (GEP) of cancer cells, human HT-12 bead array chips (Illumina, San Diego, CA, USA) were used. Methods for obtaining gene expression array data were previously reported (42, 43). Biological triplicates of DLBCL, BL, and Raji cell lines were used for the array, while six biological replicates were used for SUDHL-4 and SUDHL10. The data were corrected through normalization of the housekeeping genes with subsequent application of quantile normalization. Statistically significant genes were determined by applying a one-way ANOVA with an adjusted Bonferroni correction with a false discovery rate <0.05. Genes associated with NK ligands were compiled through a comprehensive search from literature. The overlap of genes from NK ligands with the significantly regulated genes was determined and heat map was generated using R. The raw expression data from these experiments are available at NCBI Gene Expression Omnibus database, with following identifier: GSE102930.

### Cell-Mediated Cytotoxicity Assay

CD56^+^ NK cells and lymphoma cells were cocultured at Effector to Target (E:T) ratios ranging 1:1–10:1 in low serum containing Lymphocyte Growth Medium LGM3 (Lonza) for 4 h in U bottom 96-well cell culture plates. Supernatants were collected to determine percent cell death based on GAPDH release from dying cells, using AcellaTox-Glo assay (Cell Technology, Fremont, CA, USA) and protocols as described by the manufacturer.

### Microfluidic Device Fabrication and Droplet Generation

The microfluidic devices were fabricated by standard soft-lithography protocols (31, 44–46). The device design was formulated using CAD (CAD/Art Services, Bandon, OR, USA) and printed on a transparency photomask (Fine Line Imaging, Colorado Springs, CO, USA). The design was transferred to clean silicon wafers *via* UV photolithography utilizing a negative photo resist SU-8 2100 (MicroChem, Newton, MA, USA), which was spin-coated on the wafers to obtain a layer of 150 µm height. The wafers served as master templates for elastomeric device fabrication. The prepolymer poly(dimethylsiloxane) (PDMS) (Sylgard 184, Dow Corning, Midland, MI, USA) was mixed with the silicone elastomer curing agent at 10:1 ratio (w/w), dispensed over the wafer, degassed, and cured for 12 h at 65°C. The PDMS layer containing the design network was then peeled from the wafer and separated into individual devices. Microscope slides were subjected to plasma oxidation for 30–60 s and bonded with the PDMS devices by heating at 90°C for 10 min.

Each inlet of the device was connected to individual syringes containing aqueous (i.e., cell suspension in media) or oil-based fluids through Tygon Micro Bore PVC Tubing of the following dimension: 0.010” ID, 0.030” OD, 0.010” wall (Small Parts Inc., FL, USA). The device was treated with Aquapel glass treatment (Aquapel, Pittsburg, PA, USA) for 15 min, then flushed with air immediately before experiments. The syringes were operated by individually programmable syringe pumps (Harvard Apparatus, USA). The oil to aqueous flow rates were generally maintained at a ratio of 4:1 to obtain optimal droplet sizes. The oil phase consisted of Fluorinert^®^ FC-40 (Sigma, St. Louis, MO, USA) supplemented with 2% w/w surfactant (008-FluoroSurfactant, Ran Biotechnologies, Beverly, MA, USA).

### Cell Viability Studies

Cell viability in droplets was determined by Live/Dead Viability/Cytotoxicity assay reagents (Life Technologies, Carlsbad, CA, USA). The final concentration of Calcein AM (live-cell indicator) and ethidium homodimer-1 (EthD-1, dead cell indicator) was maintained at 2 and 4 µM respectively. Calcein AM was detected by time-lapse microscopy at excitation/emission: 494/517 nm. EthD-1 was read at 528/617 nm. The proportion of live cells was calculated as a ratio of the number of live cells to the total number of cells and expressed as “Cell Death.” For co-encapsulation studies, SUDHL10 cells or patient-derived primary lymphoma cells were labeled off-chip with Calcein AM for 30 min at 37°C. The labeled cells were washed twice to remove excess cell trackers. The NK cells were left unlabeled. The two cell suspensions were loaded in separate syringes at an initial concentration of 1.5 million/mL.

### Inhibition of Secretion

1.5 million NK cells were pre-treated with Brefeldin for 2 h (GolgiPlug, BD Biosciences, San Jose, CA, USA) as per manufacturer’s recommendation (1 µL of Brefeldin for 1 mL cell suspension). Brefeldin was also added to the final cell suspension in the syringe to continue the treatment in the microfluidic droplets. In the study requiring Brefeldin and Monensin treatment, Monension (BD Biosciences) was added as per manufacturer’s recommendation. Ethylene glycol tetraacetic acid (EGTA) stock solution was prepared by dissolving EGTA in distilled water and adding 2 M NaOH to adjust the pH to 7.4. EGTA was diluted in growth media to a final concentration of 2 mM and added to the cell culture media for 1.5 h prior to co-encapsulation in droplets.

### Cytokine and Perforin ELISA

Perforin secretion was measured using Human Perforin ELISA kit from Abcam (Cambridge. MA). Human interferon gamma (IFN-γ) and tumor necrosis factor (TNF-α) secretion by NK cells were measured by Quantikine ELISA kits (R&D Systems, Minneapolis, MN, USA) as per manufacturer’s instructions. Conditioned media (CM) was collected from NK cells maintained at a concentration 1 × 10^6^ cells/mL, or NK and SUDHL10 cell mixed cultures where each cell type was kept at 0.5 × 10^6^ cells/mL. Cell debris was removed by centrifugation. Briefly, 100 µL of standard IFN-γ, TNF-α, or perforin control or cell culture supernatant were added to the antibody-coated microplate strips and incubated at room temperature for 2 h. The wells were washed thrice with the assay buffer. 200 µL of horseradish peroxidase-conjugated antibodies were added to each well for further incubation of 2 h. The wells were washed repeatedly and incubated with a combination of hydrogen peroxide and tetramethylbenzidine for 30 min. The reaction was stopped with 2N sulfuric acid and the plate read at 450 nm with a correction readout set to 540 nm.

### Image Acquisition, Processing, and Statistical Analysis

The phase/fluorescent images of cells in droplets were captured using Zeiss Axio Observer.Z1 Microscope (Zeiss, Germany) equipped with a Hamamatsu digital camera C10600 Orca-R2, 10×–40× objectives and standard FITC/DAPI/TRITC filters. The microfluidic device containing cell-encapsulated droplets was maintained in a humidified microscopic stage-top incubator at 37°C and 5% CO_2_ for the duration of the experiment. All time-lapse images were obtained by automated software control. The array was scanned to identify locations containing 1:1 effector: target ratio and the specific *x*-, *y*-, and *z*-positions were programmed in the Zen imaging program (Zeiss). Images of these locations were obtained every 5 min for a total period of 6 h. Image processing and analysis was done with ImageJ (http://rsb.info.nih.gov/ij/), Microsoft Office Excel 2010, and Origin Pro software. Fluorescent intensity of the cells at every time point was analyzed by selecting the region of interest (i.e., the cell body) and measuring mean intensity in ImageJ. Normalized fluorescent intensity (N.I) for each cell was calculated as a ratio of fluorescent intensity at every time point with respect to fluorescent intensity at the initial time. Contact periods were defined as cells forming visible conjugates for at least two consecutive time points. All periods of association and dissociation were counted for each cell and represented as percentage of total cells analyzed. NK-mediated cytolysis of target cells was characterized by the ≥80% loss of Calcein AM fluorescence from the target cells. Target death was further verified by membrane rupture and blebbing (31, 33). Killing time for contact-dependent target death was defined as the time elapsed from the initiation of contact to loss of fluorescence (as described above). Killing time for contact-independent cell death was calculated from the start of the imaging period to the target death. All statistical analysis was performed using non-parametric two-sided Mann–Whitney *U* test; *p* value < 0.05 was considered statistically significant.

## Results

### Upregulation of NK Cell Ligands and Population-Level NK Activity in Lymphoma Cells

**Figure 1:**
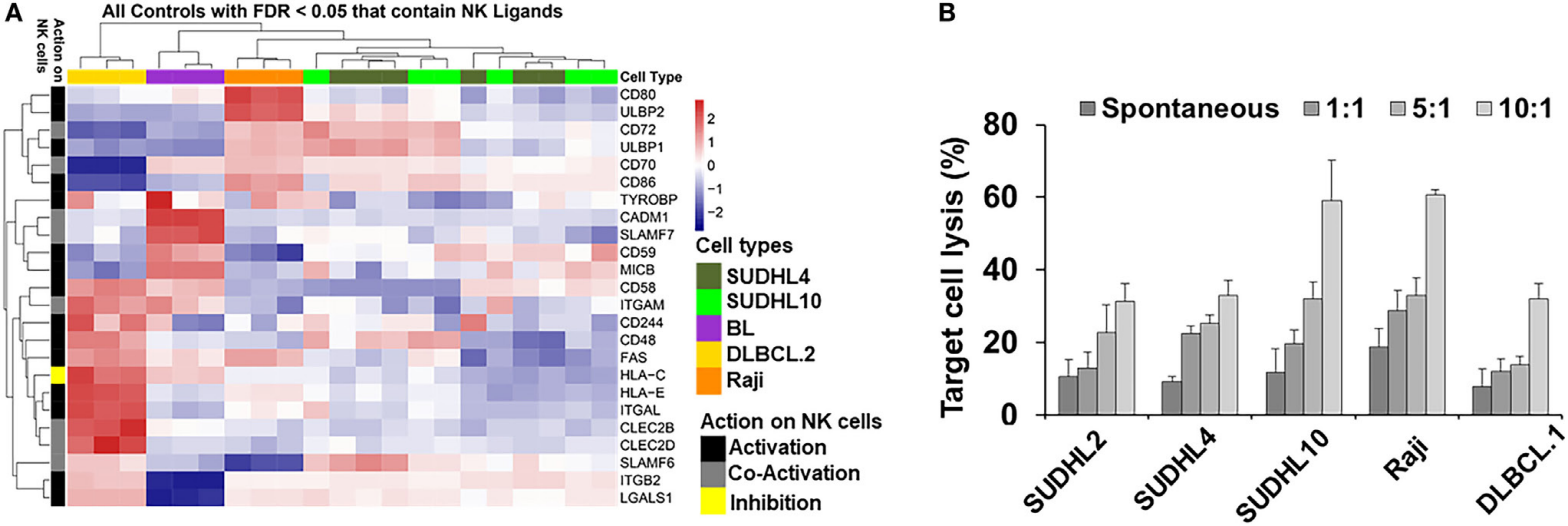
Natural killer (NK) ligands in lymphoma and cytoxicity. **(A)** Heatmap represented by hierarchical clustering show signature of gene expression patterns related to NK cell regulatory ligands in lymphoma cells and primary tumors. **(B)** Bar graph represents cell-mediated cytotoxicity as percent target lysis in lymphoma target cells cocultured with increasing ratio of NK cells. Data represented as mean lysis ± SD of three replicates. Diffuse large B cell lymphoma (DLBCL).1 and DLBCL.2 represents primary DLBCL cells obtained from two different patients. BL indicates primary Burkitt lymphoma cells from one patient.

Spontaneous detection and potent killing of lymphoma cells by CD56^+^ NK cells is strongly dependent on the presence of NK-activation ligands in the target/tumor cells. We performed GEP by microarray and determined expression levels of NK regulatory ligands in a panel of lymphoma cell lines representing various subtypes of lymphoma and primary tumors (germinal center b-NHLs: SUDHL-4 and SUDHL10; BL: Raji). Based on the analyses from GEP (Figure 1A), we noted upregulation in expression of various NK-activating ligands, but in a non-homogenous manner. Raji cells showed increased expression of NK-activation ligands (e.g., CD80, CD86, and ULBP2) in comparison with SUDHL-4 and SUDHL10 cells. The latter two lines depicted comparable NK-activation ligand expressions (e.g., ULBP1, CD72, CD48, and SLAMF6) though with sporadic variations in the expression of certain genes (including but not limited to TYROBP, CD80, and ULBP2). There was also some heterogeneity in the expression of NK cell regulatory ligand within same cell line evaluated from independent replicates; for e.g., SUDHL-4 cells from different experiments (included in GEP analysis) showed variations in patterns of NK regulatory ligand expression. Previous studies have shown that pattern of NK regulatory ligand expression could be inconsistent and subject to local influences, such as cytokines or mitogenic signaling. This can alter transcription and expression of NKG2D ligands, MICA, MICB, and ULBP1 in host cells and influence overall NK cell activity (47, 48).

Genome-wide expression profiling findings from NK regulatory ligands in the primary b-NHL cells (i.e., DLBCL.2 and BL) and b-NHL cell lines (i.e., Raji, SUDHL-4, and SUDHL10) appeared to be divergent with minimal overlap. Hierarchal clustering of the GEP heatmap showed that Raji, SUDHL-4, and SUDHL10 cells clustered together with a fairly conserved pattern of increased expression in CD72, ULBP1, CD70, and CD86 (Figure 1A). Primary cells DLBCL.2 and BL were clustered together with increased expression of CADM1, SLAMF-7, CD59, and MICB observed in BL primary cells; increased expression of CD58, ITGAM, FAS, and HLA-C in DLBCL.2 and BL primary cells; and increased expression of CD48, HLA-E, ITGAL, CLEC2B, CLEC2D, SLAMF6, ITGB2, and LGALS1 in DLBCL.2 cells.

Next, we investigated the relationship between divergent molecular signatures of NK cell regulatory ligand expression in the b-NHL cell lines with NK-specific functional cytotoxicity. IL-2-activated CD56^+^ NK cells were cocultured with target cells at effector-target (E:T) ratios 1:1 to 10:1 using GAPDH release as an indirect indicator of cell death (Figure 1B). At 1:1 E:T ratio NK-mediated target cell death was similar to spontaneous target cell lysis. Target cell lysis increased at higher E:T ratios for all cell types but were most pronounced for SUDHL10 and Raji cells. Despite upregulation of NK-activating ligands in SUDHL-4 cells, maximal cell death observed in this line was 32% at 10:1 E:T.

Taken together, these data suggest that genetic profiles may not be the sole determinant of functional response. It has been postulated that the nature of physical interactions between target and NK cells play critical roles in defining NK functional diversity, while regulatory signals or associated transcriptional programs are secondary factors (49). Therefore, our next step was to monitor dynamic interactions of NK cells with target b-NHL cells to determine NK cell activity.

### Lymphoma Cell Lysis by Contact-Dependent Mechanisms in Droplets

**Figure 2:**
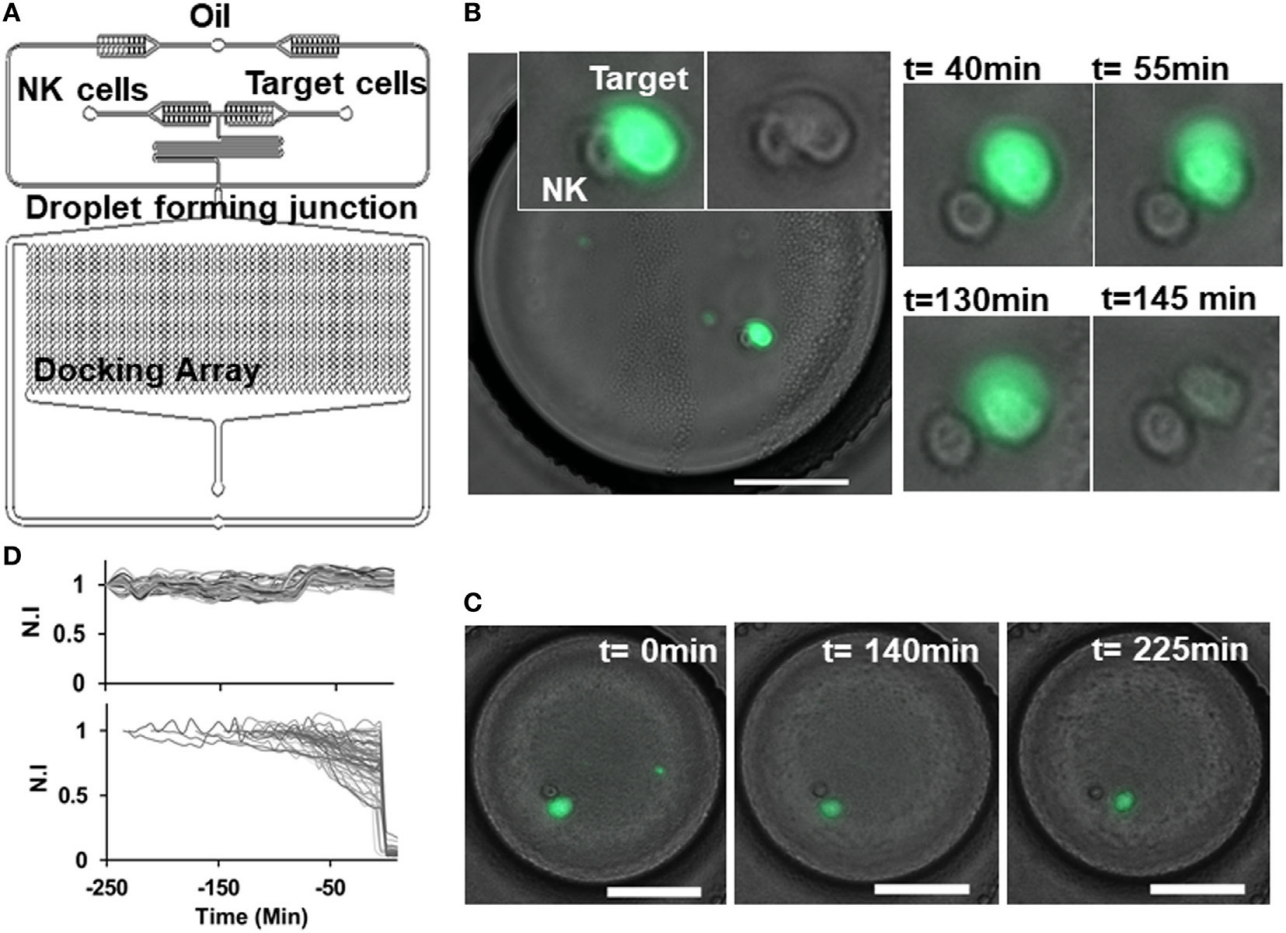
Natural killer (NK) cell dynamics in droplets. **(A)** Schematic of droplet microfluidic platform indicating NK cell and target cell channels and droplet docking array. **(B)** Contact between NK (unlabeled) and target SUDHL10 (Calcein AM labeled) cell in droplet. Loss of target cell viability was observed at 145 min. **(C)** Target cell detaches after contact with NK cell and remains viable. Scale bar: 50 µm. **(D)** Normalized fluorescent intensity (N.I) profile of live target cells (upper panel) and dying target cells (lower panel).

We developed a droplet-based cytotoxicity assay to quantify features of dynamic cell–cell interactions and determine NK cell-mediated cytolysis of target cells at single-cell level. CD56^+^ NK cells were co-encapsulated with target lymphoma cells in picoliter-volume droplets in an integrated microfluidic docking array (31, 46). NK cells were incubated through one inlet of the device and target lymphoma cells through the other inlet in a single-step cell loading process (Figure 2A). The two cell types do not make contact in the short serpentine segment due to laminar flow of the aqueous stream, restricting contact initiation in droplets. Droplets containing cells or cell pairs were generated robustly at the flow focusing zone and trapped in the docking array for live imaging and analysis (Figures 2A–C). The diameter of the droplets generated was maintained at 90 µm, resulting in droplet volumes of 380 pL. The docking array allowed trapping of 1,000–4,000 droplets per experiment. Cell densities of each cell suspension were kept at 1.5 million cells/mL to reduce the number of droplets containing multiple cells of each type, since the co-encapsulation of heterotypic cell pairs follows Poisson probability and can yield droplets consisting of 0–3 cells of each type. For the purposes of NK-mediated cytotoxicity analysis, droplets containing a 1:1 E:T ratio were monitored for up to 6 h at intervals of 5 min (Figures 2B,C).

Given the efficacy of NK cells in killing SUDHL10 cells in the population-based assay, we selected this cell line for further analysis in microfluidic droplets. We labeled the target SUDHL10 cells with Calcein AM off-chip to measure NK cell-mediated target cytolysis (31, 33). Since Calcein loading and release from cells is dependent on cell type, with some cells exhibiting inherently greater levels of Calcein release (21, 50), we assessed the retention of this viability tracker in SUDHL10 cells (Figure 2D). In the absence of effector cells, SUDHL10 target cells showed continued presence of Calcein AM in the droplets. NK-mediated target cell lysis and death was defined by a sharp decrease in fluorescent intensity of Calcein due to leakage from the damaged cell membrane (Figure 2D).

**Figure 3:**
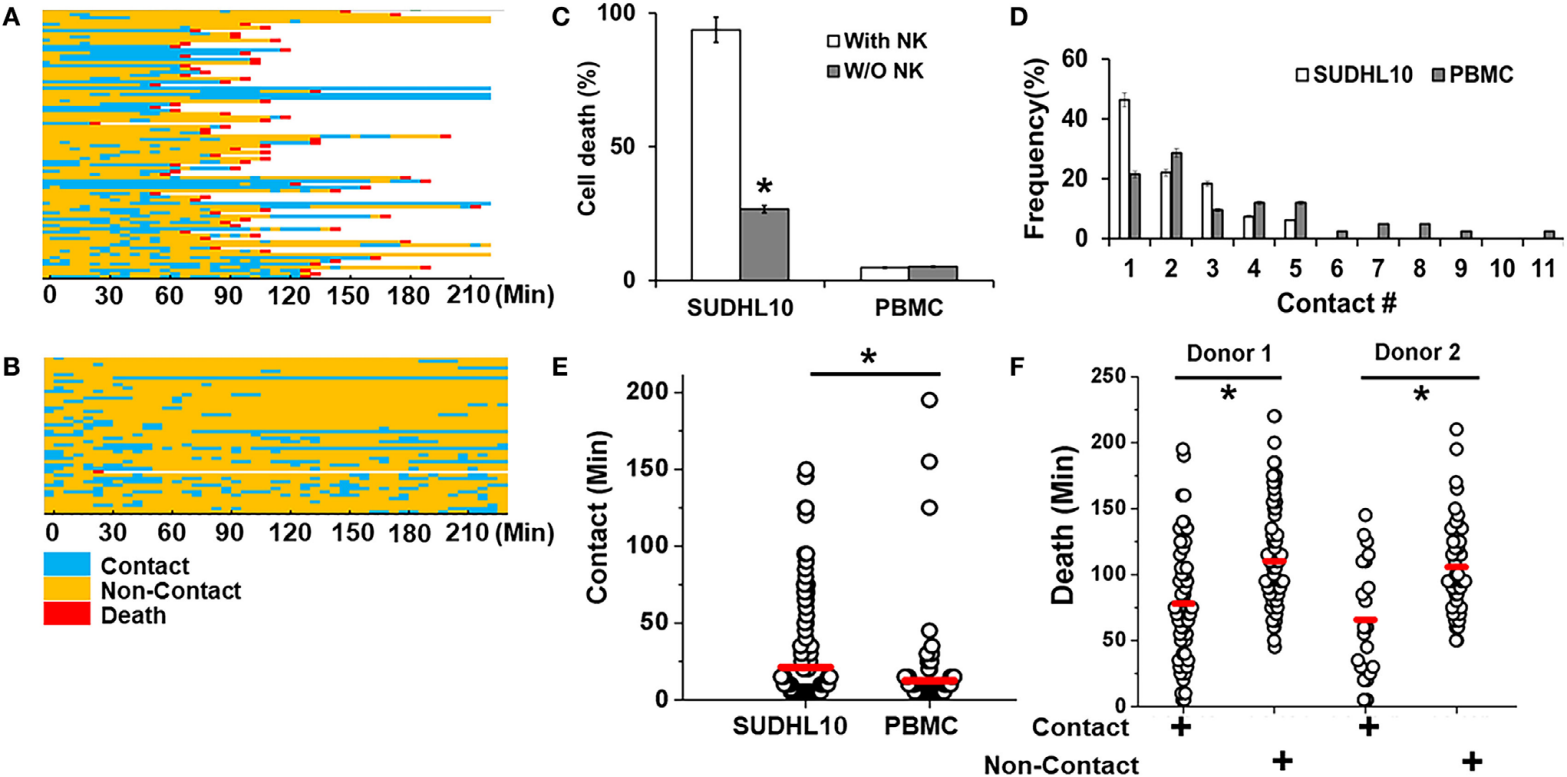
Quantitative analysis of NK cell interactions in droplets. Heat maps showing periods of contact (blue), non-contact (yellow), and death (red) during NK cell interaction with **(A)** SUDHL10 cells and **(B)** peripheral blood monocytes (PBMC). **(C)** Death of SUDHL10 (*n* = 265) and PBMC (*n* = 67) in droplets mediated in the presence or absence of NK cells. *indicates *p* < 0.005. Data show mean ± SD of two independent experiments. **(D)** Frequency of contacts made by SUDHL10 and PBMC with NK cells. **(E)** Distribution of contact duration between NK and target cells (SUDHL10 = 82; PBMC = 49) in droplets (* indicates *p* = 0.008). **(F)** SUDHL10 cell death due to contact-dependent and -independent mechanisms in droplets (*p* < 0.0005). Healthy NK cells were obtained from two different donors. Cell pairs assessed for Donor 1: 175; Donor 2: 90. The mean value of each distribution in **(E,F)** is indicated by the red line.

We imaged the dynamics of single NK cells interrogating SUDHL10 lymphoma cells in droplets to assess the heterogeneity of interaction and subsequent cytolysis (Figure 3A). Primary NK cells showed high viability in droplets [97 ± 2% in two experiments (159 and 197 cells respectively)]. Co-encapsulation of CD56^+^ NK cells with SUDHL10 cells resulted in 94 ± 4% target cell death, whereas control SUDHL10 cells (without NK cells) showed significantly lower cell death, *p* < 0.005 (Figure 3C; Video S1 in Supplementary Material). In contrast, peripheral blood monocytes (PBMC) showed 4–5% death with or without NK cells in droplets (Figures 3B,C). Both PBMCs and SUDHL10 cells established contact with NK cells, with some cell pairs showing multiple sequential contacts (Figures 3A–D). The contact durations were heterogeneous for both cell types; the mean contact duration for NK-SUDHL10 cell pairs was 20 min, and for NK-PBMC cell pairs 12 min (*p* < 0.005) (Figure 3E). The average contact duration for SUDHL10 cells that made 2–5 contacts with NK cells was less (18 min) than that of the cells that made single contacts (37 min) with NK cell (*p* < 0.05), although individual cells varied (as shown in Figures 3A,B). The difference between the total periods of contact between cell pairs making 2, 3, 4, and 5 contacts was insignificant. Barring 4% (out of 67 cells assessed) of PBMCs that made very long (>100 min) contacts, the trend varied for PBMCs that yielded an average of single and multiple cell contact periods of 12 and 11 min, respectively.

SUDHL10 cell death times varied slightly depending upon the donor NK cell population, as expected (e.g., 66–78 min). However, there was not a significant difference in the distribution of contact durations or death times between the two donors (Figure 3F). Separating the cells that made single contacts versus multiple contacts, we established that death of single-contact SUDHL10 cells occurred at an average of 57 min, while that of multiple-contact SUDHL10 cells occurred at 92 min (*p* < 0.0001). This indicated that interrupted contacts led to a delay in death times. Unlike SUDHL10 cells, PBMCs were not lysed whether the cells made single or repeated contacts with NK cells. PBMCs are not considered susceptible to NK cell-dependent cytolysis. Collectively, these data suggested that the droplet-based cytotoxicity assay was capable of demonstrating NK cell-specific lysis at the single-cell level.

### Target Cell Cytolysis in Droplets by Contact-Independent Methods

**Figure 4:**
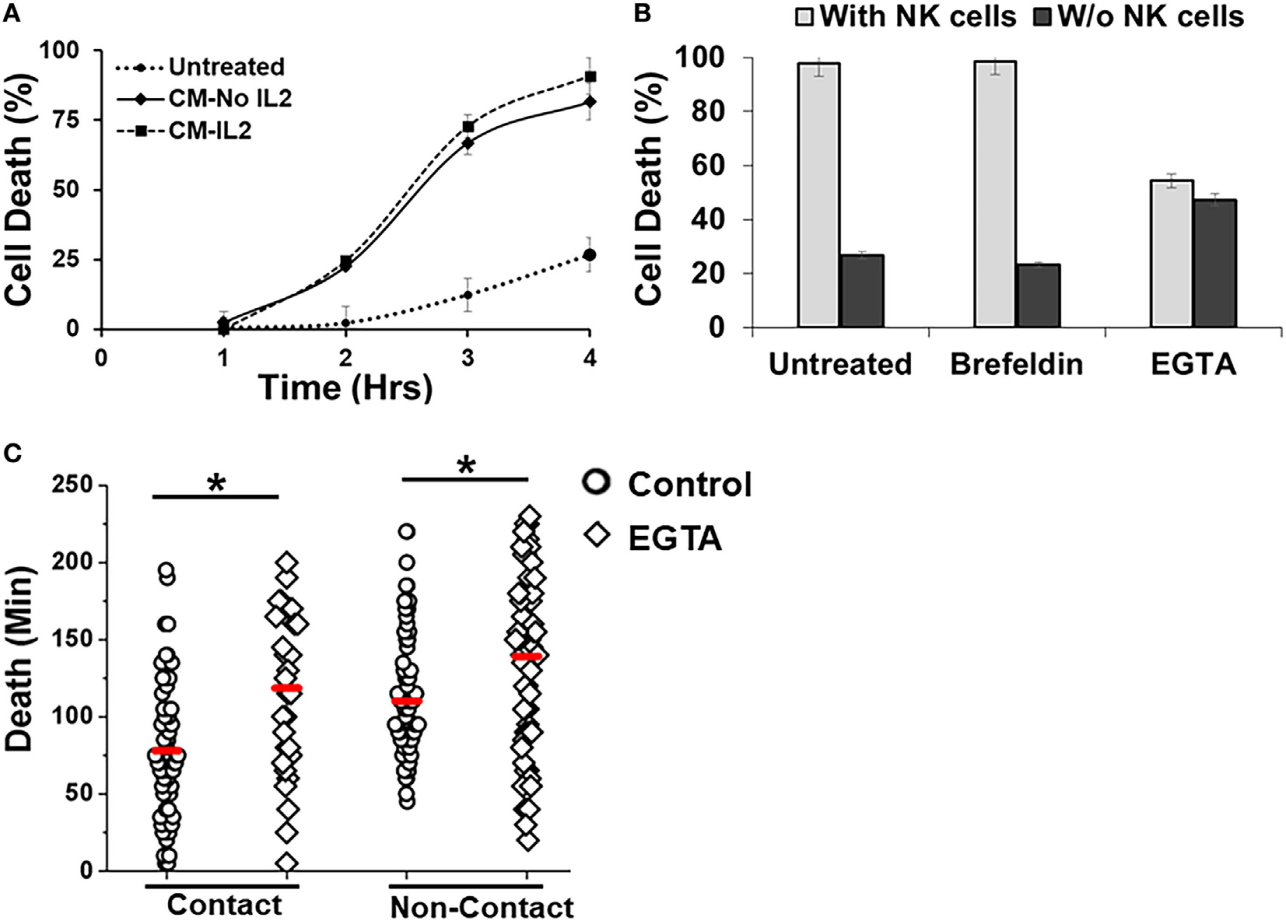
Effect of inhibitors on Natural killer (NK)-mediated cytotoxicity. **(A)** SUDHL10 cells were incubated in droplets with conditioned media (CM) from IL-2-treated (CM-IL-2; *n* = 199) or untreated (CM-No IL-2; *n* = 169) NK cells. Control SUDHL10 cells were incubated in growth media (*n* = 236). Target cell death was determined in the absence of NK cells over 4 h. Data show Mean ± SD of two independent experiments. **(B)** SUDHL10 cell death in droplets, with or without NK cells, in the presence of inhibitors Brefeldin (*n* = 144) and ethylene glycol tetraacetic acid (EGTA) (*n* = 148). Untreated: *n* = 175 cells. Data show mean ± SD of two independent experiments. **(C)** Time required for EGTA-induced SUDHL10 cell death compared to untreated cells (EGTA: *n* = 148; untreated: *n* = 175 cells). The mean value of each distribution is indicated by the red line. * indicates *p* < 0.0002.

The droplet-based cytotoxicity imaging assay allowed observation of cell death of target cells without establishment of direct contact with NK cells (Figure 3F). In droplets without visible contact between co-encapsulated NK and SUDHL10 cells, we observed 96 ± 4% death of SUDHL10 cells (92 of 93 and 61 of 66 droplets in two experiments). This occurred irrespective of donor NK cells. PBMCs that did not make contact with NK cells were not killed by this mechanism (0 out of 23 cells). Contact-independent target cell death has not been reported in single-cell cytotoxicity assays previously (33–35). While we cannot completely rule out spontaneous death of SUDHL10 cells in the droplets, this loss of viability is significantly higher than SUDHL10 cell death in the absence of NK cells (i.e., 26 ± 1%). Comparing the lytic times of the target cells that underwent contact against those that did not, we observed that contact-independent cell death occurred at significantly delayed times, which persisted in NK cells obtained from different donors (Figure 3F). To further investigate this response, we encapsulated SUDHL10 cells with conditioned media (CM) from NK cells (Figure 4). We observed that CM from IL-2-stimulated NK cells as well as non-stimulated NK cells were capable of inducing cytotoxicity in SUDHL10 cells even in the absence of NK cells (Figure 4A). This suggests that NK cells can rapidly kill SUDHL10 cells without immunological synapse formation *via* contact-independent mechanisms.

**Figure 5:**
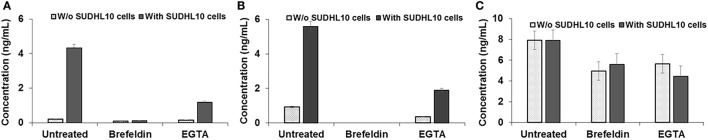
Cytokine secretion by natural killer (NK) cells. **(A)** IFN-γ, **(B)** TNF-α, and **(C)** Perforin secretion by NK cells, cocultured with or without target SUDHL10 cells, in the presence of inhibitors Brefeldin and EGTA. Mean ± SD of three replicates.

Natural killer cells are known to secrete cytokines that lead to programmed death in target cells (51). Given the confined volume of microfluidic droplets, it is possible that cytokines rapidly accumulate and reach a threshold for maximal killing within the duration of the experiment. We determined that the presence of target cells increased secretion of IFN-γ and TNF-α from NK cells (Figure 5). Treatment with Brefeldin (52), a well-known inhibitor of cytokine release, resulted in a very prominent decrease of IFN-γ and TNF-α secretion both in the presence and absence of NK cells (Figures 5A,B). However, treatment with Brefeldin did not decrease target cell death in droplets for either contact-dependent or -independent cell death (Figure 4B). Likewise, a combination of Brefeldin and Monensin had no effect in modification of target cell death (97 ± 1%, data obtained from 144 and 125 cells in two experiments) in droplets.

Next, we assessed whether perforin inhibition could affect NK-mediated cytolysis. Reagents such as concanamycin A inhibit perforin; however, it is also known to increase death of both effector and target cells (53). Therefore, we chose to use EGTA to analyze the role of the perforin/granzyme pathway in target cell killing. EGTA is a calcium chelator and is known to inhibit calcium-dependent perforin polymerization (53, 54). Treatment with EGTA has been previously shown to inhibit NK cell-mediated cytotoxicity (53, 55). We observed that EGTA decreased overall SUDHL10 cell death compared with untreated SUDHL10 cells without affecting the extent of contact between the two cell types (Figure 4B). EGTA treatment also significantly increased the time required for lysis of target cells (Figure 4C). Additionally, we determined that NK cells constitutively secrete perforin (Figure 5C). The presence of target cells did not alter this secretion. Brefeldin and EGTA both reduced the levels of perforin in NK-CM but did not eliminate it. Thus, we postulate that calcium-dependent cellular processes, including perforin secretion but not cytokines, are likely responsible for NK-mediated target cell death in the absence of direct cell–cell contact.

### Quantification of Patient-Derived Primary Lymphoma Cell Response in Droplet-Based Cytotoxicity Assay

In population-level cytotoxicity studies, we observed that CD56^+^ NK cells lysed lymphoma cells derived from a DLBCL patient at 10:1 E:T (Figure 1B). Clinical cell samples are usually available in small numbers; thus it may not be possible to determine the response of these cells to various therapies using conventional platforms that require large cell concentrations and high E:T ratios. However, it is of interest to estimate the response of tumor cells to a proposed treatment *a priori* so as to determine the best course of treatment for any particular patient in an effort to advance the concept of personalized medicine. We specifically applied our microfluidic approach to determine the susceptibility of different subtypes of b-NHL cells to NK cell lysis. However, due to limited cell numbers single-cell analysis could only be conducted once with primary lymphoma cells.

**Figure 6:**
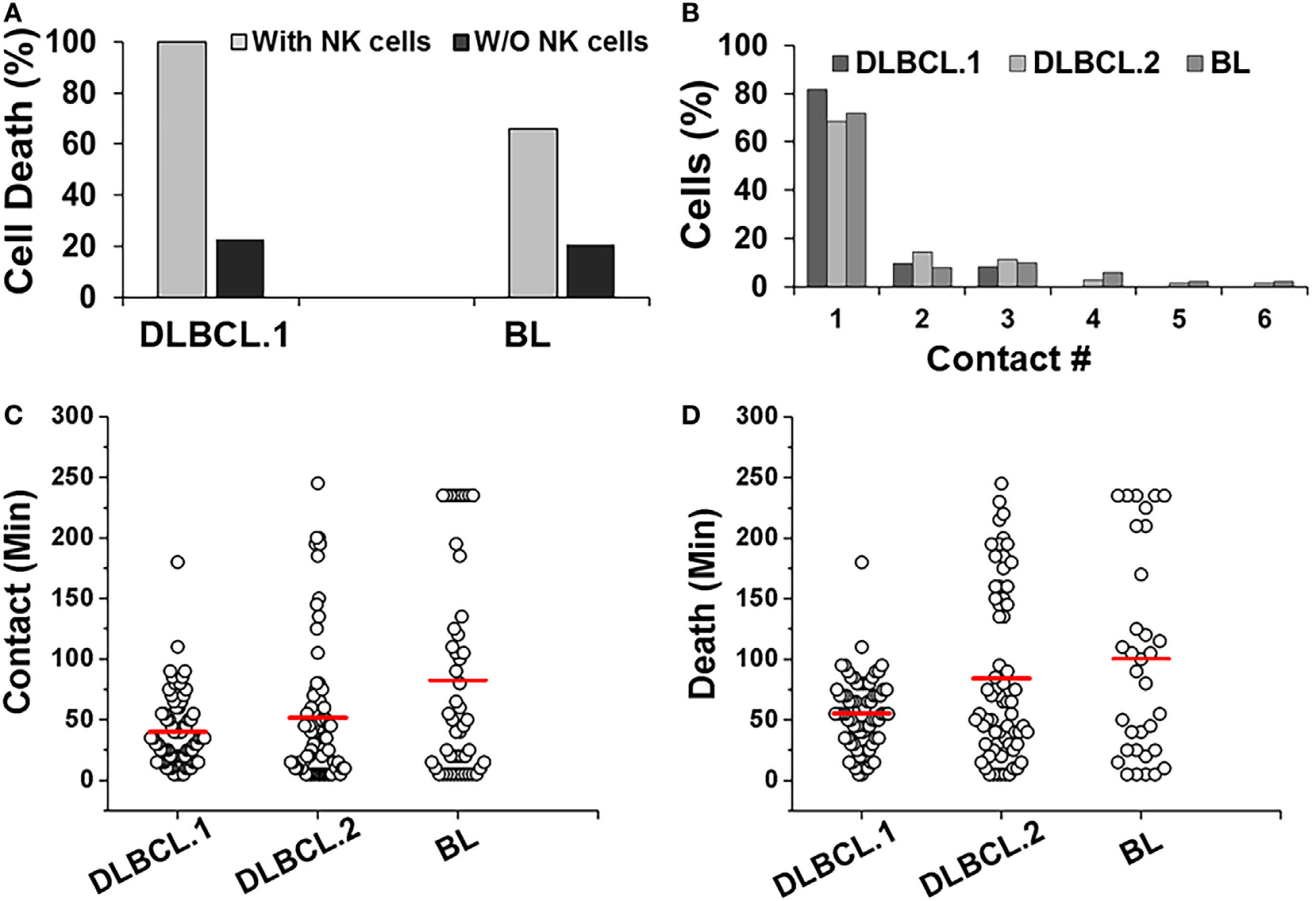
Response of patient-derived lymphoma cells in droplets. **(A)** Death of primary Diffuse Large B cell Lymphoma (DLBCL) and Burkitts Lymphoma (BL) cells in droplets in the presence (with NK cells) or absence (w/o NK cells) of NK cells. DLBCL.1: *n* = 150; BL: *n* = 95. **(B)** Serial contacts made by NK cells with DLBCL.1, DLBCL.2, and BL cells in droplets. **(C)** Duration of contacts made by DLBCL and BL cells. Mean values are indicated in red. **(D)** Primary cancer cell death due to contact-dependent mechanisms in droplets. Mean values are indicated in red. Primary cells were obtained from one patient per disease subtype and data reported from one experiment per cell type. **(A,B)** % values shown for pooled data.

Primary DLBCL cells from two patients (referred as DLBCL.1 and DLBCL.2) survived robustly in the droplets, but were more susceptible to killing by NK cells (100%). In contrast, 66% of patient-derived BL cells underwent death in the droplets (Figure 6A). All three cell populations primarily made single contacts with NK cells in droplets (Figure 6B). Overall, the two primary DLBCL cell lines demonstrated very similar contact durations with NK cells but more variability was observed in primary BL cells (Figure 6C). 98% of DLBCL.1 cells were killed in 2 h. DLBCL.2 cell death times were more distributed, resulting in a delay of the mean death time for the subset (Figure 6D). The death times of primary BL cells were also highly diverse. All cell types were subject to death by contact-dependent as well as -independent means and no statistical difference could be observed between the two subsets in each disease category.

Genome-wide expression profiling studies revealed that common NK-activation markers such as ULBP1/2, CD70, 72, 80, and 86, were downregulated in both DLBCL.2 and BL (Figure 1A). However, there were also clear differences in the molecular signatures of the two cell types. Among other genes, Fas and CD244 were upregulated in DLBCL.2 cells compared with BL, which could result in stronger NK activation by DLBCL.2 cells in comparison with BL and consequently rapid DLBCL.2 death.

### Comparing Efficacy of Primary NK Cells with NK-92 Cell Line

**Figure 7:**
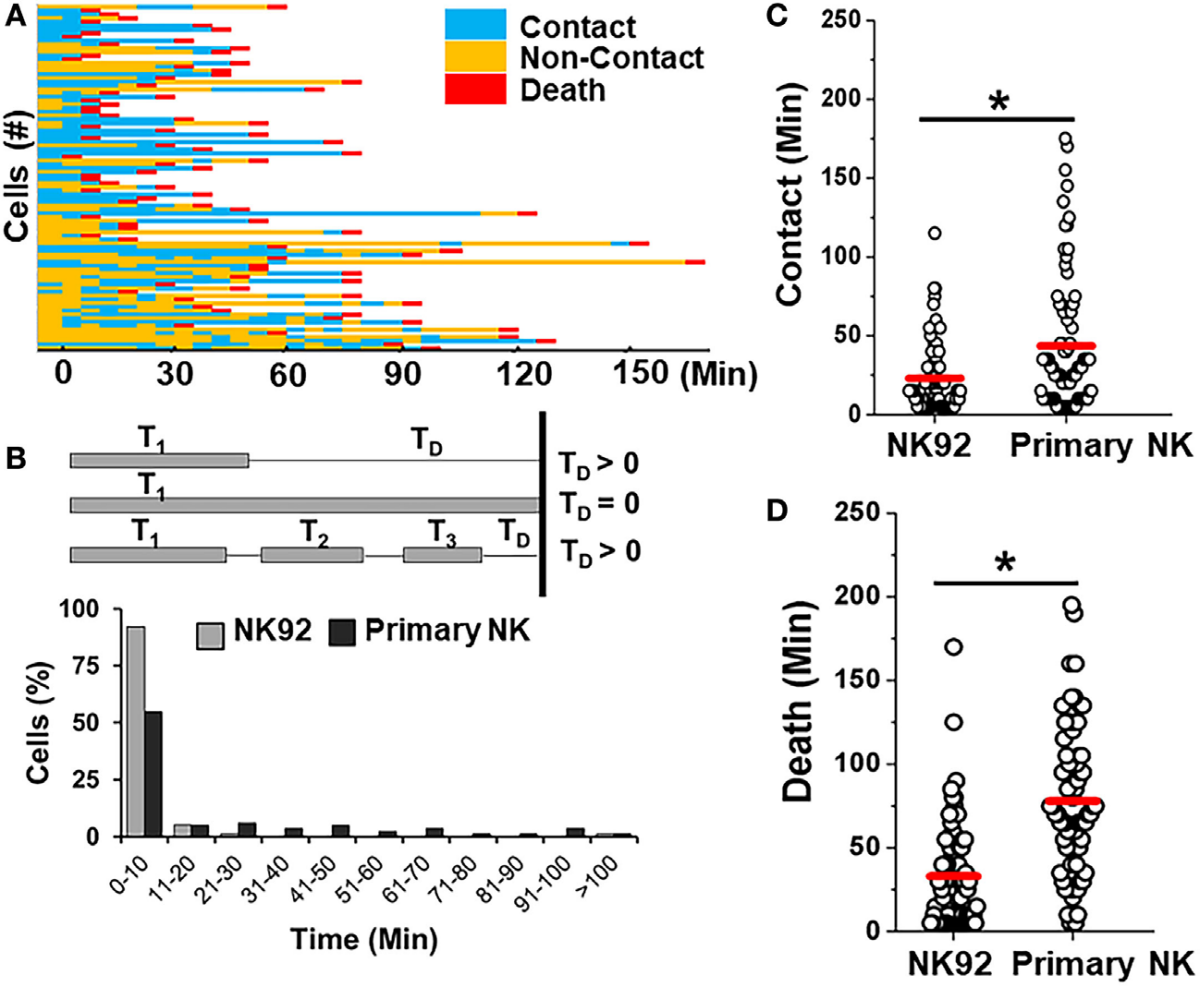
Comparison of killing efficiency of primary natural killer (NK) cells with NK92 cells. **(A)** Representative heat map showing periods of contact (blue), non-contact (yellow), and death (red) during NK92 cell interaction with SUDHL10 cells. **(B)** Schematic illustrating delay time (T_D_) calculation and T_D_ detected for SUDHL10 cells from pooled data. **(C)** Distribution of contact duration between different types of NK cells and SUDHL10 target cells in droplets (* indicates *p* < 0.0008). **(D)** SUDHL10 cell death mediated by NK92 cell line and primary CD56^+^ NK cells. The mean value of each distribution in **(C,D)** is indicated by the red line. Cell pairs evaluated for NK92: 77; Primary NK: 76 from two independent experiments.

Primary allogeneic NK cells are typically limited in supply as NK cells constitute only 10% of circulating lymphocytes. A few NK cell lines have been established in the last decade to circumvent the challenges associated with autologous or allogeneic NK cell therapy (56). NK92 cells have been characterized in preclinical models extensively and shown promising results in four phase I trials worldwide for different cancers (56). Here, we determined the feasibility of detecting differences between interactions of primary NK cells and an NK cell line (Figure 7). We co-encapsulated SUDHL10 lymphoma cells with NK-92 cells, which express CD56 molecules and have been known to consistently demonstrate potent antitumor activity (56). Both primary NK cells and NK92 cells showed high levels of target cell kill in droplets (Figures 3A and 7A). However, our microfluidic single-cell cytotoxicity assay allowed us to detect distinct characteristics exhibited by the two cell types. In contrast with primary NK cells, higher (twofold) number of contacts initiated by NK-92 cells ended in death of target cells without detachment from the immune conjugate (Figure 7B). The total time of contact between each NK-target cell pairs was significantly shorter for NK92 cells (Figure 7C). While both types of cells made single and multiple contacts, NK-92 cell contacts resulted in faster killing of target cells (Figure 7D). Altogether, these findings highlighted that NK-92 cells were more effective in lysing target cells compared with primary NK cells.

## Discussion

Direct physical interactions occurring between NK and target cells have the potential to determine NK cell effector outcome in the short run and the fate of NK cell in the long run (49). Quantifying the nature and response of single NK cells in a defined environment is useful for illustrating the functional and mechanistic diversity in NK cell interactions. To this end, we developed a droplet microfluidic approach to assess dynamic effector–target cell interaction that is particularly suitable for evaluating non-adherent cell types, such as NK and T cells (31). We investigated transient and stable interactions between NK and b-NHL cell lines as well as primary b-NHL cells to determine the feasibility of analyzing clinical-grade lymphoma cell cytotoxicity with this bioassay.

Aggressive b-NHLs are generally treatable, but there remains a significant need to identify more targeted and non-toxic therapeutic agents as well as associated biomarkers that predict enhanced activity in the treatment of b-NHL patients. Cellular immunotherapy, including NK cell-based therapies, are highly promising treatments in NHL (56–58). Our gene expression analyses predicted that the increase in expression of NK-activation ligands in lymphoma cells is favorable for NK cell antitumor activity. In addition to known NK-activating ligands such as UL16 binding proteins (ULBP), we also determined the expression of molecules shown to play key roles in NK cell cytotoxicity, particularly in the context of B cell lymphomas, such as CD80, CD86, CD70, and CD72 (59–63). However, the functional efficacy of differential gene expression on NK cell sensitivity can be hard to gauge due to microenvironmental regulations and inconsistency between gene and mRNA expression levels (64, 65). SUDHL-4 and SUDHL10 cell lines had comparable expression of NK ligands ULBP1, CD72, CD48, and SLAMF6, but our population-based cytotoxicity analysis showed that SUDHL10 cells have higher sensitivity to NK cells compared with SUDHL-4 cells. Over-expression of SLAMF-7 may become inhibitory if EAT-2 co-receptor is absent, thereby modulating cell responses differentially (66). Thus, functional studies are essential to better understand heterogeneity in effector responses. Our microfluidic analysis suggests that b-NHL cell lines are susceptible to cytotoxicity mediated by CD56^+^ NK cells *via* contact-dependent and -independent mechanisms. The results from three patient-derived b-NHL cells were also in accordance with this finding, although it needs to be corroborated with larger number of samples.

CD56^+^ NK cells were capable of killing SUDHL10 cells by contact-dependent mechanisms at 1:1 E:T in our single-cell cytotoxicity assay at greater extent compared to population-level assays (10:1 E:T). Thus, the sensitivity of the microfluidic assay exceeded that of a conventional assay in this specific target cell line. We surmise that increasing the proximity of effector and target cells enhanced the possibility of a higher kill at lower E:T ratio. While further studies are required to confirm this response in multiple target cell types, our preliminary investigations show similar increases in cytotoxic effect in myeloma and additional lymphoma lines in microfluidic droplets (unpublished observation).

The live-cell imaging aspect of this microfluidic bioassay further allowed us to assess the dynamics of cell–cell interaction at single-cell level. Our study showed that the mean contact period between NK and SUDHL10 cells was 20 min, shorter than reported values of 65 min in previous NK cell studies (33, 34). This could be attributed to different target cell lines tested in each study. NK cells are known to contact multiple targets, but our assay demonstrated multiple contacts between the same cell pair. It is unclear if these NK cells were not sufficiently activated to deliver a lethal hit following first contact with the target cell. Some studies have shown that NK cells integrate activating signals resulting from contact with spatially separated ligands (13, 67). While this effect is primarily observed when NK cells encounter multiple cells, it is feasible that similar effects can occur if an NK cell encounters distinct ligand groups on the same target cell surface. We further detected that the interrupted contacts between E–T cell pairs led to a delay in the death of target cells. The average duration of contacts made by cells that underwent multiple contacts was much lower than mean single contact period. Therefore, we conclude that shorter contact periods were not sufficient to deliver lytic hits to lymphoma cells. Previous reports also suggest the possibility of correlation between rapidity and strength of NK cell response (33). Contact-dependent killing by NK cells is mediated largely by perforin-dependent mechanisms. In this study, NK cells were found to secret perforin. However, perforin-independent pathways, such as Fas/Fas ligand and TNF-related apoptosis-inducing ligand (TRAIL) could also contribute to lymphoma cell cytotoxicity. Fas expression was observed in SUDHL10, suggesting that this molecule may play a role in mediating SUDHL10 death.

An interesting finding in our study was the death of target cells without visible contact with NK cells. Furthermore, we observed death of target cells in NK cell-CM in droplets. We determined that this killing was unaffected by the secretion of cytokines IFN-γ and TNF-α. Cytokine secretion pathways are distinct from the pathways regulating secretion of cytotoxic granules that contain perforin and granzyme (68). Cytokine secretion inhibitors, such as Brefeldin and Monensin, do not inhibit the exocytosis of existing granules but may differentially block new cytotoxic granules. Recent reports suggest that NK cells are capable of killing target cells through constitutive secretion of exosomes containing perforin and Fas Ligand (69). Both resting and activated NK cells release exosomes that appear to target tumor cell lines and activated immune cells (69). NK cell exosomes do not kill resting immune cells such as PBMCs, which may explain the lack of PBMC death in subsets of cells that did not make contact in our study. Perforin was found to be secreted by NK cells irrespective of the presence of target cells and despite treatment with cytokine secretion inhibitors. We believe that perforin secretion, potentially in exosomes, is responsible for contact-independent target cell death in microfluidic droplets. Additionally, this effect is regulated at least partially by calcium-dependent signaling as calcium chelator treatment significantly reduced NK-induced target cell death (70). This has been observed previously in T cells where EGTA inhibited T cell cytotoxicity (53). EGTA inhibits perforin pathway indirectly by blocking perforin polymerization. Our studies showed that it inhibited cytokine secretion as well. Another potential mechanism of contact-independent cell death may be the presence of Fas Ligands in NK-secreted exosomes; however, EGTA may not be a suitable reagent to distinguish perforin- and Fas-based cell-mediated cytotoxicity (54).

Our microfluidic cytotoxicity assays were also found suitable for rapid analysis of primary lymphoma cell death. To the best of our knowledge, no study has previously evaluated the dynamic interaction of NK cells with NHL cells at the single-cell level. While the possibility of a dynamic, sensitive cytotoxicity assay at low E: T ratio has great benefit in conserving clinical samples, it was practically challenging to validate single-cell findings in this study due to the limited availability and quality of primary cancer cells. We further determined the gene expression profile of primary DLBCL and BL cells and observed differential upregulation of NK-activating ligands in these cells. It must be noted that neither the NK nor the target cells used in these experiments are genetically homogenous, as they were obtained from different donors. Thus, the death of lymphoma cells observed in our assays could occur due to KIR-HLA mismatch, which is known to trigger NK cell activity. Conversely, expression of inhibitory KIR receptors on NK cell and its interaction with MHC-I self antigen expressed on target cells is known to inhibit NK cell activity against target cells. NK-92 cells, which lack KIR expression, show shorter contact with target SUDHL10 cells, followed by rapid death. Further investigations on such receptor interactions will assist in relating the individual phenotype associated with single-cell interactions, in samples obtained from multiple donors.

In conclusion, we demonstrated that the microfluidic droplet array platform provides an effective and robust technique to evaluate dynamic interactions of immune and immunotherapeutic (NK-92) cells with bNHL cells through functional phenotyping. It provided a rapid and quantitative assessment of NK cell cytotoxicity at single-cell level and was capable of resolving differences in transient interactive features of various cells (primary NK vs. NK-92), which may be underestimated in conventional studies. This approach enables studying both early activation events as well as delayed effector functions, and will be complementary to existing bulk analytical tools in revealing heterogeneity in individual cell responses. It may be further leveraged to analyze changes in morphology and functionality of modified NK cells (e.g., NK-based therapeutics) as well as to delineate the effect of immunomodulatory drugs *via* multiple mechanisms (e.g., ADCC, CDC).

## Author Contributions

SS and PS designed and conducted microfluidic experiments. DR and SPurvey conducted cytotoxicity analysis. DR and AB conducted gene microarray analysis. SS and SPotdar performed the acquisition, analysis, and interpretation of the data. SS wrote manuscript with substantial inputs from DR. AE and TK substantially contributed to the conception of the work and revised it critically for important intellectual content. All the authors approved the final version of the manuscript.

## Conflict of Interest Statement

The authors declare that the research was conducted in the absence of any commercial or financial relationships that could be construed as a potential conflict of interest.

## Supplementary Material

Video S1 Time-lapse of natural killer (NK)-SUDHL10 cell interaction and SUDHL10 cell death. NK cells are unlabeled, SUDHL10 cells labeled green.
